# Evidence for the long-distance transport of ticks and tick-borne pathogens by human travellers to Texas, USA

**DOI:** 10.1093/jtm/taaf032

**Published:** 2025-04-18

**Authors:** Michael S Allen, Rebecca J Kilgore, Yan Zhang, Megan T Williams, Sabrina N White, Pete D Teel

**Affiliations:** Tick-Borne Disease Research Laboratory, Department of Microbiology, Immunology, and Genetics, University of North Texas Health Science Center, 3400 Camp Bowie Blvd., Fort Worth, TX 76017, USA; Tick-Borne Disease Research Laboratory, Department of Microbiology, Immunology, and Genetics, University of North Texas Health Science Center, 3400 Camp Bowie Blvd., Fort Worth, TX 76017, USA; Tick-Borne Disease Research Laboratory, Department of Microbiology, Immunology, and Genetics, University of North Texas Health Science Center, 3400 Camp Bowie Blvd., Fort Worth, TX 76017, USA; Tick-Borne Disease Research Laboratory, Department of Microbiology, Immunology, and Genetics, University of North Texas Health Science Center, 3400 Camp Bowie Blvd., Fort Worth, TX 76017, USA; Tick-Borne Disease Research Laboratory, Department of Microbiology, Immunology, and Genetics, University of North Texas Health Science Center, 3400 Camp Bowie Blvd., Fort Worth, TX 76017, USA; Department of Entomology, Texas A&M AgriLife Research, 370 Olstead Blvd., College Station, TX 77843, USA

**Keywords:** Tick-borne diseases, international travel, *Ixodes ricinus*, *Borrelia afzelii*, vector-borne infections, *Amblyomma*, *Ixodes*, emerging pathogens

## Abstract

**Background:**

The incidence of tick-borne diseases in the USA has surged in recent years, with >50 000 cases reported from an estimated half-million cases annually. While domestic vectors are well characterized, the role of human travel in transporting exotic ticks and pathogens remains poorly understood.

**Methods:**

We analysed 4808 submissions of ticks removed from individuals to the Tick-Borne Disease Research Laboratory in Texas, USA, from 2004 to 2024. Tick species were identified phenotypically or through DNA-based sequencing, and pathogens (*Borrelia* spp., *Rickettsia* spp., *Ehrlichia* spp., *Anaplasma phagocytophilum* and *Babesia microti*) were detected using molecular assays. Submitter travel histories were cross-referenced with known tick geographic ranges to identify long-distance transport.

**Results:**

We documented cases of intercontinental, international and domestic interstate transport of ticks by human travellers. Four Ixodes ricinus ticks—originating from Europe—were transported to Texas; one tick carried *Borrelia afzelii*, a Lyme disease pathogen not endemic to North America. Central and South American *Amblyomma* species were repeatedly imported, raising concerns for pathogen transmission and tick establishment. Domestic travellers also carried *Ixodes scapularis* ticks infected with *Borrelia burgdorferi* sensu stricto, *Babesia microti* and *A. phagocytophilum* from Lyme-endemic regions of the USA to Texas, along with non-native species *Ixodes pacificus*, *Dermacentor andersoni* and *D*ermacentor* occidentalis* from endemic areas in the western United States.

**Conclusions:**

Long-distance transport of ticks by travellers represents an underrecognized pathway for the global spread of ticks and tick-borne pathogens. Clinicians should consider travel history in tick-borne illness diagnostics. Enhanced surveillance, public education and travel screening are critical to mitigating these risks.

## Introduction

Tick-borne diseases represent a rapidly growing vector-borne health threat in the USA, accounting for 75% of all reported vector-borne illnesses.^1^ The number of tick-borne disease cases in the continental United States has been increasing, led by Lyme disease (*Borrelia burgdorferi*), which alone accounted for an estimated ~ 476 000 cases annually during the years spanning 2010–2018.^2^ These cases were predominantly in the Northeast, Upper Midwest and West Coast; however, the geographic range of ticks and their associated pathogens in the USA and abroad continue to expand due to many factors.^3^^,^^4^

The unintentional human transport of arthropod vectors of disease is a critical yet often overlooked public health concern that is known to occur on a global level yet is frequently lacking supporting data. While the global transport of mosquitoes has garnered significant attention, particularly following the 1999 introduction of *Aedes aegypti* and West Nile virus to the USA,^5^ the movement of ticks remains underappreciated. The recent introduction of the Asian longhorned tick, *Haemaphysalis longicornis*, has reignited concerns related to the spread of ticks and tick-borne diseases,^6^ along with fears of purposeful introduction of disease vectors as acts of bioterrorism.^7^ Despite the increasing recognition of this threat, data on the long-distance transport of ticks, particularly those attached to human travellers, remain sparse.

Despite several publications and reviews discussing the importation of ticks via animals across various contexts,^8–10^ there are comparatively few published reports of human transportations of ticks over long distances and usually reporting individual instances.^11–16^ With billions of airline passengers and extensive global travel by land and sea each year,^17^ the hidden risk of humans inadvertently transporting ticks is likely far greater than currently recognized, posing a silent but significant threat to public health worldwide.

To address this gap and uncover the currently hidden risks of long-distance transport of ticks and the pathogens they carry via attachment to human travellers, we conducted analysis of 20 years of tick submission reports submitted to the Tick-Borne Disease Research Laboratory (TBDRL) at the University of North Texas Health Science Center (UNTHSC) in cooperation with the Texas Department of State Health Services. By examining these reports, we aimed to identify instances of human transport of ticks and uncover patterns of interstate, international and intercontinental tick transport and evaluate the potential risks of tick and tick-pathogen introduction to new regions.

Our findings provide crucial insights into the hidden dynamics of tick-borne disease transmission and emphasize the need for enhanced surveillance and preventative measures to mitigate emerging risks. Through a comprehensive retrospective review of tick sample submissions to the TBDRL over a 20-year period (2004–2024), we report multiple instances of interstate, international and intercontinental transport of ticks and associated pathogens by human travellers. These findings reveal an underappreciated pathway for tick and pathogen dispersal, raising significant concerns for public health and highlighting the global reach of human-assisted tick movement.

## Methods

The Texas Department of State Health Services (DSHS), in partnership with the UNTHSC TBDRL, accepts submissions of ticks removed from humans for free pathogen testing from Texas state citizens. Tick samples are first submitted to the Texas DSHS along with a completed form, which includes the question blank ‘Geographic location where tick was likely acquired (describe geographic location, including Texas county OR other state/country—e.g. Zilker Park, Austin, Travis County)’. The complete form can be accessed online at https://www.dshs.texas.gov/sites/default/files/IDCU/health/zoonosis/forms/human/Texas-Tick-Fillable-Submission-Form-103122.pdf. During the sample submission process, tick species is first determined phenotypically by state entomologists at the Texas Department of State Health Services laboratory in Austin, Texas. Samples are subsequently sent to the TBDRL for pathogen testing, along with the completed submission form.^18^^,^^19^ When phenotypic identification of tick species was uncertain, TBDRL staff conducted DNA-based identification of tick species by amplification and sequencing of the tick mitochondrial 16S rRNA gene, followed by sequence comparison to the NCBI database using BLAST.^19^ All ticks were tested for the presence of pathogens within the genera *Borrelia*, *Rickettsia* and *Ehrlichia* as previously described.^18^^,^^19^ Beginning 7 December 2023, testing for *B. burgdorferi* was changed to a qPCR-based methodology, and pathogen targets were expanded to include additional qPCR tests for *Babesia microti* and *Borrelia miyamotoi,*^20^ as well as for *Anaplasma phagocytophilum* beginning 1 January 2024.^21^ All submitted samples consisted of individual ticks, the vast majority of which belong to the family Ixodidae, with the exception of 17 sample submissions of *Otobius megnini*, family Argasidae*.* All specific samples discussed herein were adult ticks unless otherwise noted.

To identify incidents of transport of ticks from outside of the state, we first assessed all submissions in which the identified tick species was not endemic to the state. Next, we evaluated metadata contained in the form associated with each tick submission, including patient-reported, suspected geographic origin of the tick, stated history of recent travel and local address of the submitter, to retrospectively assess potential incidents of long-range transportation of human-biting ticks. All tick sample submissions with reported origins that included a site outside of Texas were initially flagged for further review. During that review, the following selection criteria were applied to all samples. Tick sample submissions with a stated origin reported as being from outside of the state were considered as ‘Probable’ cases of transport provided the tick species is known to occur in that region. Tick submissions with a stated origin as being either from Texas or somewhere outside of the state were considered as ‘Possible’ cases of tick transport, again provided that the tick species is known to also occur in both regions. Only in cases when the submitted tick species was not native to the state or the submitted tick was identified as carrying a pathogen not endemic to Texas but known to be present in the reported possible geographic origin of the sample were considered as evidence of transport from outside of the state. Tick sample submissions with reported origins inside the state of Texas were not considered further unless the species of the tick was considered not endemic to the state. It should also be noted that no attempts were made to specifically confirm the submitter’s mode of transportation for ticks submitted for testing. While it is reasonable to conclude air travel was the means of transport for transoceanic travellers, ground and in some cases ship transportation must also be considered for interstate and international transport within North America, as discussed below. Additionally, tick geographic origin was reported by submitters and not independently verifiable. However, as stated, only cases in which the known geographic range of the tick species coincided with the reported origin of the tick were included in this report. Despite these restrictions on selection criteria, several examples of interstate, international and intercontinental transport of ticks were identified.

## Results

All tick samples submitted to the TBDRL from Texas residents over the years spanning 2004–2024 were evaluated for this study. In total, 4808 individual tick sample submission reports were reviewed. Of these, 4263 (88.66%) were reported as originating from Texas, 292 (6.07%) submissions reported an origin outside of Texas (including 15 reporting more than one possible state), 49 (1.0%) submissions reported origins as Texas or a second location and 204 (4.25%) submissions listed no origin location. Ixodid ticks identified by the TBDRL originated from 11 countries outside of the USA and 37 of the 50 states in the USA (Table 1). Of the intercontinental cases, four instances of transport of ticks from Europe to Texas were identified. All four of these cases involved transportation of *Ixodes ricinus*, from areas in Central and Western Europe (Table 2). The specific cases included *I. ricinus* ticks reportedly acquired in the Czech Republic (2016), France (2021), Germany (2022) and Belgium (2024). Among these, the sample from Germany was also found to be positive for the Lyme disease agent *Borrelia afzelii*. This bacterium is the most common Lyme disease agent in Europe and not known to be present in North America,^22^ further supporting a European origin of the tick and underscoring the potential risk of introduction of new disease agents to the USA.

**Table 1:** Reported geographic origin of ticks (Ixodidae) submitted to TBDRL between 2004 and 2024 and assessed in this study

US State	Alabama^(0/1)^, Arkansas^(48/7)^, California^(5/0)^, Colorado^(6/1)^, Connecticut^(2/0)^, Delaware^(2/0)^, Florida^(1/0)^, Georgia^(2/1)^, Illinois^(1/1)^, Indiana^(3/0)^, Kansas^(4/0)^, Kentucky^(9/0)^, Louisiana^(6/1)^, Maine^(2/2)^, Maryland^(5/0)^, Massachusetts^(5/1)^, Michigan^(2/1)^, Minnesota^(2/2)^, Mississippi^(2/1)^, Missouri^(15/1)^, Montana^(1/0)^, New Hampshire^(0/1)^, New Jersey^(1/0)^, New Mexico^(0/1)^, New York^(12/2)^, North Carolina^(12/0)^, Oklahoma^(81/11)^, Pennsylvania^(7/2)^, South Carolina^(1/0)^, South Dakota^(0/1)^, Tennessee^(10/3)^, Texas^(4263/0)^, Virginia^(10/1)^, Vermont^(1/0)^, West Virginia^(3/1)^, Wisconsin^(1/0)^, Wyoming^(3/0)^
North America	Belize^(2/0)^, Canada^(1/0)^, Costa Rica^(2/1)^, Guatemala^(0/3)^, Honduras^(1/1)^, Mexico^(5/0)^
South America	Ecuador^(1/0)^
Europe	Belgium^(1/0)^, Czech Republic^(1/0)^, France^(1/0)^, Germany^(1/0)^

^a^Numbers in parentheses refer to the number of samples identified by submitters as being from that geographic location, followed by the number of samples that were described as being either from Texas or a second location outside of the state (probable cases/possible cases).

**Table 2:** Specific examples of ticks (Ixodidae) associated with humans travelling to Texas from outside areas highlighted in this 2004–2024 study

Reported tick origin	Tick species	Year of submission	Pathogens detected
California, USA	*Ixodes pacificus* (f)	2008	n.d.
California, USA	*I. pacificus* (f)	2013	n.d.
California, USA	*I. pacificus* (f)	2015	n.d.
California, USA	*I. pacificus* (f)	2023	n.d.
California, USA	*Dermacentor occidentalis* (x)	2023	n.d.
Colorado, USA	*Dermacentor andersoni* (m)	2017	n.d.
Colorado, USA	*D. andersoni* (f)	2017	n.d.
Colorado, USA	*D. andersoni* (m)	2019	n.d.
Massachusetts, USA	*Ixodes scapularis* (x)	2013	*Borrelia burgdorferi* s.s.
Minnesota, USA	*I. scapularis* (f)	2016	*B. burgdorferi* s.s.
Montana, USA	*D. andersoni* (f)	2016	n.d.
Pennsylvania, USA	*I. scapularis* (f)	2022	*B. burgdorferi* s.s.
Pennsylvania, USA	*I. scapularis* (f)	2024	*Anaplasma phagocytophilum* *Babesia microti*
Virginia, USA	*I. scapularis* (f)	2024	*B. burgdorferi* s.s. *A. phagocytophilum*
Wyoming, USA	*D. andersoni* (f)	2006	n.d.
Wyoming, USA	*D. andersoni* (f)	2024	n.d.
Belgium	*Ixodes ricinus* (f)	2024	n.d.
Belize	*Amblyomma coelebs* (n)	2017	n.d.
Belize	*A. coelebs* (n)	2017	n.d.
Belize	*A. coelebs* (x)	2024	n.d.
Ontario, Canada^a^	*Dermacentor variabilis* (f)	2010	n.d.
Costa Rica or Central Texas^a^	*Amblyomma* sp. (n)	2006	n.d.
Costa Rica	*Amblyomma tapirellum* (n)	2009	n.d.
Costa Rica	*Amblyomma mixtum* (n)	2018	n.d.
Czech Republic	*I. ricinus* (n)	2016	n.d.
Quito, Ecuador	*A. coelebs* (n)	2021	n.d.
France	*I. ricinus* (f)	2021	n.d.
Germany	*I. ricinus* (f)	2022	*B. afzelii*
Guatemala^a^	*A. mixtum* (n)	2023	n.d.
Guatemala^a^	*A. mixtum* (n)	2023	n.d.
Honduras^a^	*D. variabilis* (m)	2023	n.d.
Roatan, Honduras or Texas^a^	*Amblyomma americanum* (n)	2013	n.d.
Tamaulipas, Mexico^a^	*D. variabilis* (m)	2019	n.d.

^a^Uncertain origin (m = male, f = female, n = nymph, x = undetermined, n.d. = none detected).

Ample evidence of the repeated transport of ticks from South and Central America to Texas was also identified. In 2009, an *Amblyomma tapirellum* nymph was submitted by a traveller returning to Texas from Costa Rica. In two separate instances, *Amblyomma coelebs* nymphs were submitted in 2017 reportedly from Belize, with a third *A. coelebs* sample from Belize submitted in 2024. In the last instance, further communication with the submitter revealed that the individual and two travelling companions had been clearing land in Belize. Upon return to the USA, the travel companions also reported finding ticks on their persons, but these were discarded without further reporting.^23^ Another *A. coelebs* nymph was also submitted in 2022 for testing by a traveller returning from Quito, Ecuador. Transport of this species on an airline passenger from Central America to the USA has also been reported elsewhere.^12^ Both *A. tapirellum* and *A. coelebs* are endemic to their respective reported countries of origin, and neither are thought to occur natively in Texas, providing additional support to the accuracy of the reported source locations.^24–26^

Other possible instances of the transport of ticks from Central America and Mexico to the USA. identified in this study were less clear. In one case, a submitted tick was reported as being either from Texas or from Costa Rica. In that instance, the tick was identified as an *Amblyomma* species nymph, but subsequent efforts to conclusively identify the tick species by amplicon sequencing of the mitochondrial 16S gene from this specimen failed. In another case also reportedly from Costa Rica, two *Amblyomma mixtum* nymphs were submitted. This species is found in both Costa Rica and the southernmost regions of Texas, confounding conclusive source attribution.^27^^,^^28^ Nevertheless, a nymph of the same species attached to a traveller returning to Germany from Cuba has previously been reported.^15^ In 2013, an *A*mblyoma* americanum* nymph was submitted and reported as potentially originating from either Texas or Roatan, Honduras. This species is commonly found across Texas but may also be present in Honduras,^29^ complicating attribution of the origin of this sample. Five male *Dermacentor variabilis* ticks reportedly originating from Tamaulipas, Mexico, were submitted together for testing in 2019. The range of this species spans a large portion of North America, including portions of both Mexico and Texas, and extending northeasterly into Canada,^30^ again making certain attribution of the tick origin difficult. This is also true for a *D. variabilis* tick reportedly originating from Ontario, Canada, submitted in 2010. Similarly, a patient reportedly travelling from Honduras and passing through Guatemala and Mexico to Texas submitted a *D. variabilis* tick, while two other individuals in separate incidents and both reportedly originating from Guatemala and again passing through Mexico to Texas submitted two *A. mixtum* ticks. In all these cases, the submitters reported discovering the ticks after arriving in Texas.

In addition to international transportation of ticks on travellers, we identified multiple records of ticks being transported from across the USA to Texas, including specimens reported from 37 of the 50 US states (Table 1). In many cases, the tick species can be found in both Texas and the reported origin area, leaving little to conclusively demonstrate an origin outside of Texas. However, there were several instances where this was not the finding, and we focused our attention on these cases. For example, four submissions to the TBDRL were identified as *Ixodes pacificus*, a tick native to the Pacific coast of North America.^31^^,^^32^ These four submissions were provided by different individuals, although all reportedly originated from California. In another submission from California, a *Dermacentor occidentalis* was submitted. This tick is distributed from southern California to southwestern Oregon.^33^ Samples of *Dermacentor andersoni* were identified from three submissions reportedly from Colorado, along with one each from Montana and Wyoming. *D. andersoni* has an endemic range spanning the mountainous regions of the western United States to southern Canada.^34^

Numerous submissions containing *Ixodes scapularis* have also been submitted to the TBDRL, and several of these reportedly originated from outside Texas. The range of *I. scapularis* spans most of the eastern United States, including the eastern half of Texas, and its range has been expanding northward into southern Canada^35^ However, the bacterial Lyme disease agent *B. burgdorferi* sensu stricto, for which *I. scapularis* is the principal vector, is not endemic to Texas, but is found primarily in the northeastern states comprising New England and the upper Midwest states west of the Great Lakes.^36^ Identification of *B. burgdorferi* s.s. within *I. scapularis* ticks reportedly from the northeastern and upper midwestern United States are therefore consistent with their origin and transport from those areas. Due to the high numbers of *I. scapularis* submissions originating from within Texas, additional examples of *I. scapularis* ticks reportedly originating from other states in the absence of *B. burgdorferi* s.s. (or later, *A. phagocytophilum* and/or *Babesia microti*, which are similarly rare or not reported for Texas) were considered as probable but not confirmatory evidence of interstate transport. Similarly, ticks found broadly across the USA, but which do not vector a specific, regionally endemic pathogen, were similarly limited to probable but not conclusive cases, including numerous examples of *I. scapularis*, *D. variabilis*, *Amblyomma*  *maculatum* and *A*.* americanum*. One interesting exception to this was a single case from 2021, in which an individual reported an *A. americanum* tick originating from Texas that was discovered and removed after the submitter reached Sioux Falls, South Dakota, which is thought to be outside the northernmost range of this species.^37^ This represents the only reported instance in our data of a tick originating from Texas and being transported outside of the state.

## Discussion

This study unveils compelling evidence of long-distance tick and pathogen transport facilitated by human travellers, emphasizing a critical but underappreciated risk to public health. Our retrospective analysis of 20 years of tick submissions to the TBDRL demonstrates that human movement, particularly through air travel, serves as a recurrent mechanism for tick dispersal. Instances of interstate, international and intercontinental transport were identified, including cases where imported ticks carried pathogens not endemic to the USA, such as *B. afzelii*, a major Lyme disease agent in Europe. Ticks from as many as 11 countries and 37 US states were found to be transported to Texas, underscoring the scale of this phenomenon. Importantly, many cases involved species not native to Texas, highlighting the potential for the introduction of exotic vectors and novel pathogens into previously unaffected regions.

While cumulative results of tick-pathogen testing at the TBDRL have been periodically published,^18^^,^^19^ this study is the first to systematically examine long-range tick transport and provide concrete data supporting these conclusions. The findings reveal the mostly silent yet potentially significant role of human travel in the geographic movement of ticks and their pathogens, calling for greater public health attention to patient travel history and vector-pathogen awareness.

### Comparison with previous studies

Our findings align with previous reports that highlight the role of human travel in the translocation of arthropod vectors.^12^^,^^15^ However, this study adds critical depth by providing a broader temporal scope and documenting multiple instances of tick transport over a 20-year period. Notably, we identified four cases of the *I. ricinus* transported to Texas from Europe, with one of these cases involving a tick infected with *B. afzelii*. This pathogen, previously unreported in North American wildlife, raises the possibility of novel pathogen introductions with potentially profound public health implications.

*Ixodes ricinus* is considered a competent vector of *B. burgdorferi* sensu lato, including the common genospecies *B. burgdorferi* s.s., B. afzelii and *Borrelia garinii*, as well as a number of other pathogenic bacteria (*A. phagocytophilum*, *Francisella tularensis*, *Rickettsia helvetica* and *Rickettsia monacensis*), piroplasmid parasites (*Babesia divergens* and *Babesia microti*) and viruses (tick-borne encephalitis virus, Louping ill virus and Tribec virus).^38^  *B*orrelia* burgdorferi* s.s. is the most common tick-borne pathogen identified in the USA,^36^ and *B. garinii* has previously been identified in eastern Canada^39^^,^^40^ and South Carolina.^41^ Rudenko *et al*.^41^ analysed two isolates obtained from South Carolina rodents with phylogenetic analyses of global *B. garinii* isolates including those from Canada. They concluded that the Canadian and US isolates of *B. garinii* did not have a common origin and suggested that the ancestral lineage of the Canadian isolates was European while the lineage of the South Carolina isolates was from East Asia. *B*orrelia* afzelii*, identified in the tick originating from Germany, has not previously been reported in wild animal populations in North America. The identification of these four separate cases of *I. ricinus* ticks from Europe raise particular concern given the number of pathogens the species can vector and the presence of related and potentially suitable vectors in the USA.

### Emerging risks from Central and South America

The repeated transport of *A. coelebs* and *A. mixtum* from Central America to Texas adds another layer of concern. These species, though less studied, are possible vectors for Spotted Fever Group *Rickettsia* and *Ehrlichia* species. Considerably less is known about the broader vectorial capacity of these species, raising concerns of unknown pathogen introductions. The relatively recent discoveries of Heartland virus and Bourbon virus in the USA associated with *A. americanum*^42^^,^^43^ underscore the potential for imported ticks to introduce new, uncharacterized pathogens, even in the absence of established populations. These findings highlight the gaps in our understanding of the full vectorial capacity of these species.

### Traveller infestations by ticks

Activities that bring human travellers into contact with tick-infested habitats and/or infested animals offer opportunities for ticks to attach. Ticks in the family Ixodidae (*Amblyomma*, *Dermacentor*, *Ixodes*) found in the TBDRL submissions are three-host ticks and could attach to humans as larva, nymph or adult. Discovery of a tick attached to a person is increased with tick size, thus adult ticks are most, and larvae least, likely to be discovered. In addition, the length of attachment (days) increases with each successive stage (larva to adult).

### Limitations

Several limitations pertain to the current study. First, as a passive surveillance effort, tick samples received are wholly dependent on voluntary submissions, which may be skewed by a number of factors such as demography, education levels or perceived risk. Other factors such as a possible increase in the likelihood of submission following travel to certain high-risk areas might also confound results reported here. Second, the reported origin of the tick is primarily based on the submitter’s estimation, which can be particularly difficult given that ticks may adhere for several days providing time for individuals to potentially pass through multiple geographic areas prior to discovery. Third, not all possible avenues for testing were explored. Pathogen testing did not include all possible pathogenic agents, most notably testing for viruses was not conducted, which may have provided additional insights into tick origins. Similarly, no efforts were made to further differentiate tick species into regional subgroups using genetic markers. Finally, the voluntary submission of nearly 5000 samples over a 20-year period is particularly small considering the millions of Texas residents annually engaged in national, international and transcontinental travel and potentially exposed to ticks during travel activities. There are 32 official air, sea and land ports of entry in Texas supporting domestic and global commerce (https://gov.texas.gov/uploads/files/business/PortsOfEntry.pdf) and a 1254-mile (2018-km) international border through which legal and illegal traffic add to the dynamic flow of humans with potential ‘excess baggage’ of arthropods and arthropod-borne pathogens important to human and animal health.

## Conclusions

The incidents of long-distance tick transport identified in this study significantly expand the documented evidence of human-mediated vector movement, reinforcing the notion that this represents an underreported and underappreciated public health risk. The results also highlight the risk of human-mediated travel for the potential introduction of non-native arthropod species and pathogens they may carry. The global scale of human travel necessitates greater awareness of ticks and tick-borne pathogens and the implementation of preventive measures. Educating travellers on the risks of tick exposure, improving surveillance at points of entry, and fostering international collaboration for vector surveillance and control are crucial steps towards mitigating the spread of ticks and tick-borne pathogens. The data presented here provide a foundation for developing evidence-based strategies to address the growing threat posed by human-assisted tick transport and its implications for global health. Furthermore, this study emphasizes the complementary but critical role of passive tick surveillance and reporting as essential public health measures for monitoring the transport and spread of ticks and tick-borne pathogens. These findings also underscore the need for clinicians to inquire about travel history when diagnosing febrile illnesses or suspected tick-borne diseases, as imported ticks may carry pathogens unfamiliar to the local ecosystem. Similarly, health care providers and public health officials should stress the risks of tick-borne illness when opportunities arise, such as to individuals seeking vaccines for upcoming travel. The U.S. Centers for Disease Control and Prevention (CDC) provide additional guidance for travellers for the prevention of ticks and other arthropod vectors of disease, along with information on tick removal.^44^ Individuals travelling and engaging in activities that increase the risk of exposure are cautioned to apply preventative measures and follow-up with full-body tick checks after returning indoors.^45^
